# Disorganized bone collagen fibers in early-stage type 2 diabetes mellitus and its association with adipose tissue redistribution

**DOI:** 10.1186/s41747-026-00820-0

**Published:** 2026-10-01

**Authors:** Ying Liu, Qian Meng, Shuo Chen, Xiaojian Zhang, Lei Gao, Ming Wang, Lin Bai, Xuesong Zhang, Ping Zhang, Ning Zheng, Jian Zhao

**Affiliations:** 1 Department of Medical Imaging, Hebei Medical University Third Hospital, Shijiazhuang, China; 2 Department of Radiology, Beijing Geriatric Hospital, Beijing, China; 3 Clinical & Technical Support, Philips Healthcare, Beijing, China

**Keywords:** Adipose tissue, Bone density, Bone matrix, Diabetes mellitus (type 2), Liver

## Abstract

**Objective:**

Type 2 diabetes mellitus (T2DM) compromises bone quality and biomechanical integrity, driven by impaired microstructure, suppressed turnover, and collagen abnormalities. Crucially, this fragility occurs despite normal bone mineral density and may initiate early in the disease, underscoring the need for timely skeletal assessment.

**Materials and methods:**

Twelve male Sprague-Dawley rats were randomly assigned to either the control or the T2DM group. Within 2 weeks of model confirmation, all underwent dual-energy x-ray absorptiometry, *in vivo* magnetic resonance imaging of the liver and femur, and blood collection for comprehensive biochemical and lipid profiling. Subsequently, all right femurs were harvested for micro-computed tomography, biomechanical testing, and Masson’s staining. The tibia and fibula were collected for the analysis of bone metabolism markers and advanced glycation end products (AGEs).

**Results:**

Compared with the control group, T2DM rats exhibited higher body weight from week 10 onward, along with increased fat mass and body fat percentage. Serum total cholesterol, alanine aminotransferase and aspartate aminotransferase levels were elevated. Hepatic proton density fat fraction (PDFF) was higher. No intergroup differences of serum calcium, magnesium and phosphorus, cancellous/cortical bone and biomechanical testing parameters. Femoral collagen fiber organization was impaired in T2DM rats. Procollagen type I N-terminal propeptide was upregulated, while osteocalcin relative mRNA expression was downregulated. Femoral collagen organization was strongly negatively correlated with hepatic PDFF.

**Conclusion:**

In early-stage T2DM, bone damage follows a temporal sequence (organic before inorganic) and site-specific (pronounced in cancellous bone). These changes parallel systemic metabolic disturbances, and ameliorating adipose and hepatic dysfunction may help restore bone collagen matrix homeostasis.

**Key Points:**

***Question*** How does the metabolic microenvironment shaped by adipose tissue redistribution affect bone microarchitecture in patients with early-stage T2DM?

***Findings*** In early-stage T2DM, bone damage may exhibit a temporal (organic-first) and spatial (cancellous-predominant) pattern, and coexists particularly with abnormal lipid metabolism and hepatic dysfunction.

***Relevance statement*** In early-stage T2DM, abnormal lipid metabolism and liver function may serve as a potential indicator of compromised bone quality. Therapeutic interventions aimed at ameliorating adipose and hepatic dysfunction in T2DM may help restore bone collagen matrix homeostasis.

**Graphical Abstract:**

**Figure:**
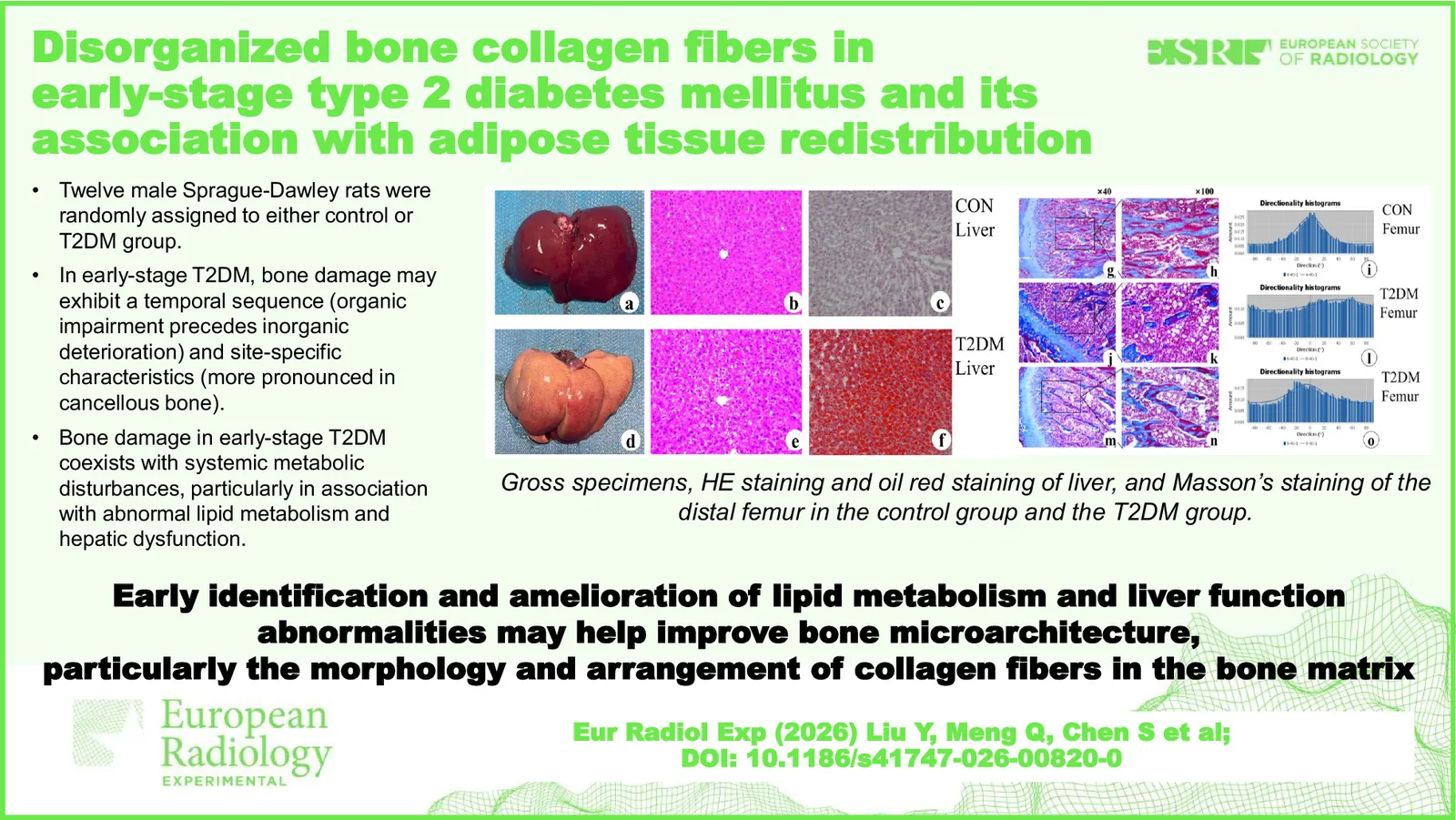

## Background

Type 2 diabetes mellitus (T2DM) is a systemic metabolic disorder characterized by adipose accumulation, insulin resistance, and chronic inflammation [1, 2]. Contemporary understanding of T2DM and its complications has evolved from a traditional “hyperglycemia-centric” perspective toward a broader framework of “systemic metabolic dysregulation.” In this context, dysfunction of visceral adipose tissue and consequent ectopic lipid deposition—such as hepatic steatosis—are recognized as early and critical events in disease progression [3]. These pathological processes lead to the release of bioactive molecules, including free fatty acids and pro-inflammatory cytokines, thereby establishing a systemic microenvironment conducive to metabolic injury. A study published in *Radiology* in 2025 demonstrated that the liver fat fraction is strongly associated with the odds of T2DM in a dose-dependent manner, an effect partially mediated by liver enzymes [4].

Beyond its well-characterized systemic and hepatic manifestations, T2DM exerts profound effects on the musculoskeletal system. Although historically the skeletal complications of T2DM were considered late-stage phenomena, accumulating evidence suggests that bone impairment may begin much earlier. A growing body of research has confirmed that bone fragility in T2DM arises from a combination of impaired bone microstructure, suppressed bone turnover, and abnormal bone matrix composition, culminating in reduced biomechanical competence [5]. A longstanding paradox in the field has been the observation of normal or even elevated areal bone mineral density (BMD) alongside increased fracture risk—highlighting the limitations of traditional bone mass-centric assessments and motivating a paradigm shift toward the concept of “bone quality” [6–8]. Bone quality encompasses all structural and material properties that contribute to bone strength beyond BMD, including trabecular microarchitecture (commonly expressed by DXA-derived trabecular bone score) [9], cortical geometry (commonly: DXA-derived hip structural analysis) [10], bone deformation under loads (commonly indicated by DXA-derived bone strain index) [11], bone turnover rate, microdamage accumulation, degree of mineralization, bone marrow microstructure [12], and the integrity of the organic matrix, particularly collagen content and spatial structure.

Consequently, the research focus has shifted from fracture outcomes to an in-depth exploration of the mechanisms underlying impaired bone quality. The organic matrix, particularly the type I collagen fiber network, is essential for maintaining bone toughness and resistance to fracture. In the hyperglycemic environment of T2DM, advanced glycation end products (AGEs) form abnormal cross-links between collagen fibers, compromising their elasticity and repair capacity [13]. This deterioration in collagen integrity is now considered a core mechanism underlying bone brittleness in T2DM. Notably, such changes may be initiated early in the disease trajectory, underscoring the need for timely evaluation of skeletal health in individuals with T2DM.

We hypothesized that the metabolic microenvironment, shaped by adipose tissue redistribution and hepatic lipid deposition, acts as a primary driver of structural and spatial disorganization in the bone organic matrix in early-stage T2DM, particularly within the collagen network. This damage may exhibit spatiotemporal specificity, occurring first in metabolically active trabecular bone, with alterations in the organic matrix preceding abnormalities in mineral deposition.

## Materials and methods

This animal study was prospective, approved by the ethics committee of Hebei Medical University Third Hospital (IRB number: Z2024-008-1, Shijiazhuang, China), and complied with animal rights declarations and regulations.

### Experimental animals and T2DM modeling

Twelve 7-week-old male specific pathogen–free Sprague-Dawley (SD) rats (mean weight = ~270 g) were purchased from Beijing Huafukang Bioscience Co., Ltd. They were housed under controlled conditions (temperature = 22 °C ± 2 °C; humidity = 60% ± 5% [mean ± standard deviation]; 12-h light/dark cycle) with ad libitum access to food and water.

**Fig. 1:**
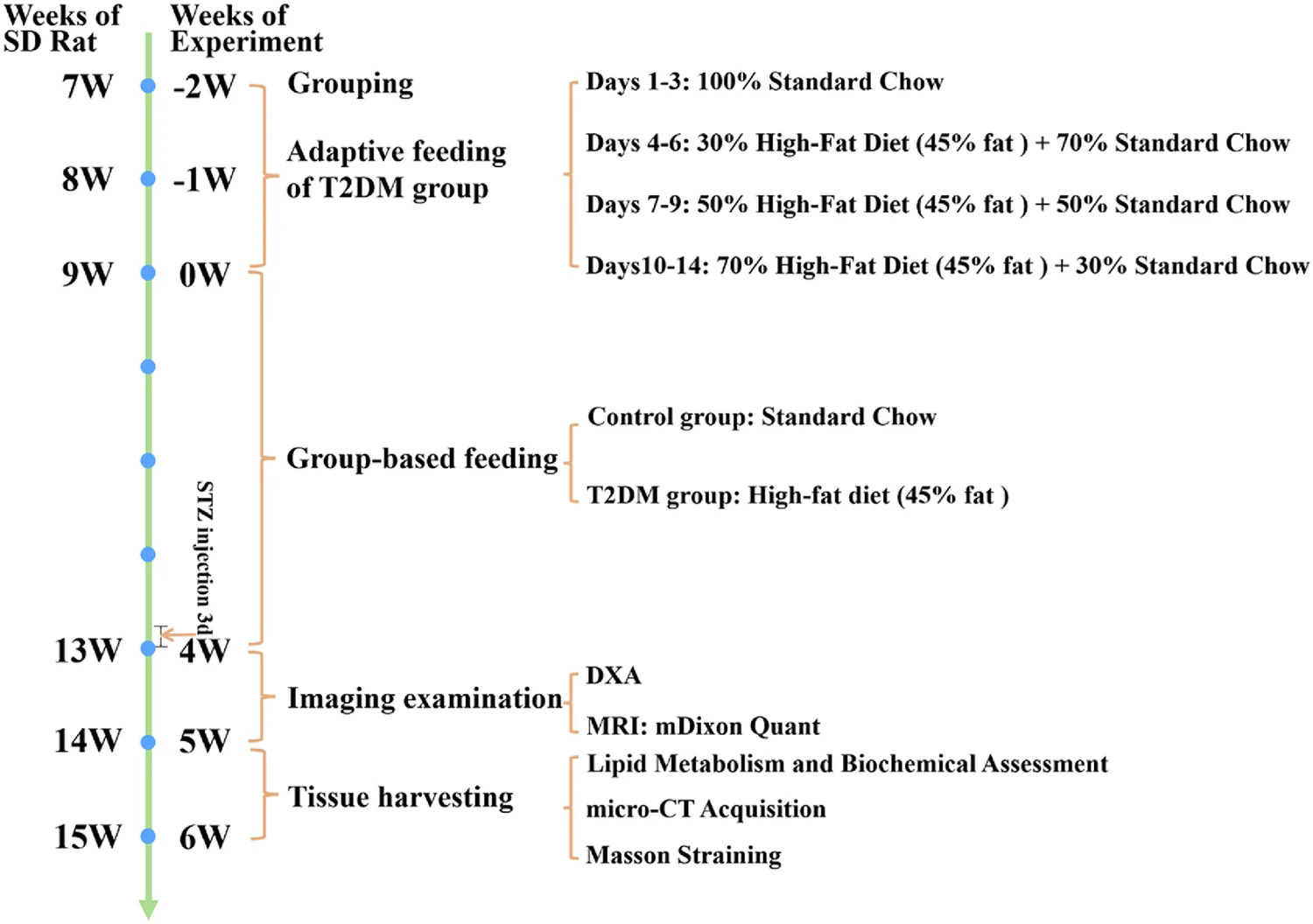
Flowchart of SD rat feeding and establishment of the T2DM model. SD, Sprague-Dawley; STZ, Streptozotocin; T2DM, Type 2 diabetes mellitus

Rats were randomly assigned to a control group or a T2DM group. The sample size of 6 per group was chosen as it is consistent with previous studies in this field [14] and was considered sufficient to detect biologically relevant differences, in line with the 3 R principle for ethical animal research [15]. After a 2-week acclimatization period, the control group received a standard diet, whereas the T2DM group received a custom-mixed diet formulated with specific proportions of high-fat and standard chow. The formal dietary intervention began at 9 weeks of age. The control group continued to receive the standard diet, whereas the T2DM group was fed a high-fat diet containing 45% fat for 4 weeks (Fig. 1).

The body weight was measured every 3 days. During the final 3 days of the fourth week, rats in the T2DM group received intraperitoneal streptozotocin injections (50 mg/kg in citrate buffer; a daily dose for 3 days; pH = 4.2), whereas those in the control group received an equivalent volume of normal saline. The principle of using streptozotocin in T2DM modeling is based on its glucose transporter type 2‑mediated selective toxicity to pancreatic β‑cells. Through low‑dose administration, streptozotocin partially destroys the islets and, in the context of insulin resistance induced by a high‑fat diet, forces the body into a decompensated hyperglycemic state. This strategy effectively balances the two core pathophysiological features of insulin resistance and impaired insulin secretion and is currently one of the gold standard animal models for studying the pathogenesis of T2DM and for drug screening [16, 17].

Fasting blood glucose levels were measured from the tail vein using a portable glucometer (Fig. 1). The T2DM model was considered successful when fasting blood glucose levels consistently exceeded 7.5 mmol/L. After confirmation of T2DM, dual-energy x-ray absorptiometry (DXA) and magnetic resonance imaging (MRI) were completed within 1 week, followed by cervical dislocation and tissue harvesting during the subsequent week (Fig. 1).

### DXA protocol and image analysis

All rats were anesthetized with isoflurane and positioned supine in a standardized manner on the scanning bed of a DXA system (InAlyzer; MEDIKORS, Seongnam, South Korea). The animals were carefully aligned to ensure that the entire body was fully contained within the scan field. Upon completion of the scan, the system software automatically generated whole-body composition data. The recorded parameters included fat mass (g), lean mass (g), and body fat percentage (%).

### MRI protocol and image analysis

Prior to MRI examination, all rats were anesthetized with isoflurane. After achieving stable anesthesia, the rats were positioned prone on the scanning bed, with the region of interest (ROI) centered within the coil. Anesthesia was maintained throughout the scanning procedure using continuous delivery of isoflurane at a constant concentration via a nose cone, while the respiratory rate and body temperature were monitored. Hepatic and femoral PDFF values were quantified using a 3.0-T MRI system (Ingenia CX; Philips Healthcare) with a 3-dimensional multi-echo chemical shift-encoded MRI sequence (mDIXON Quant; Philips Healthcare).

**Fig. 2:**
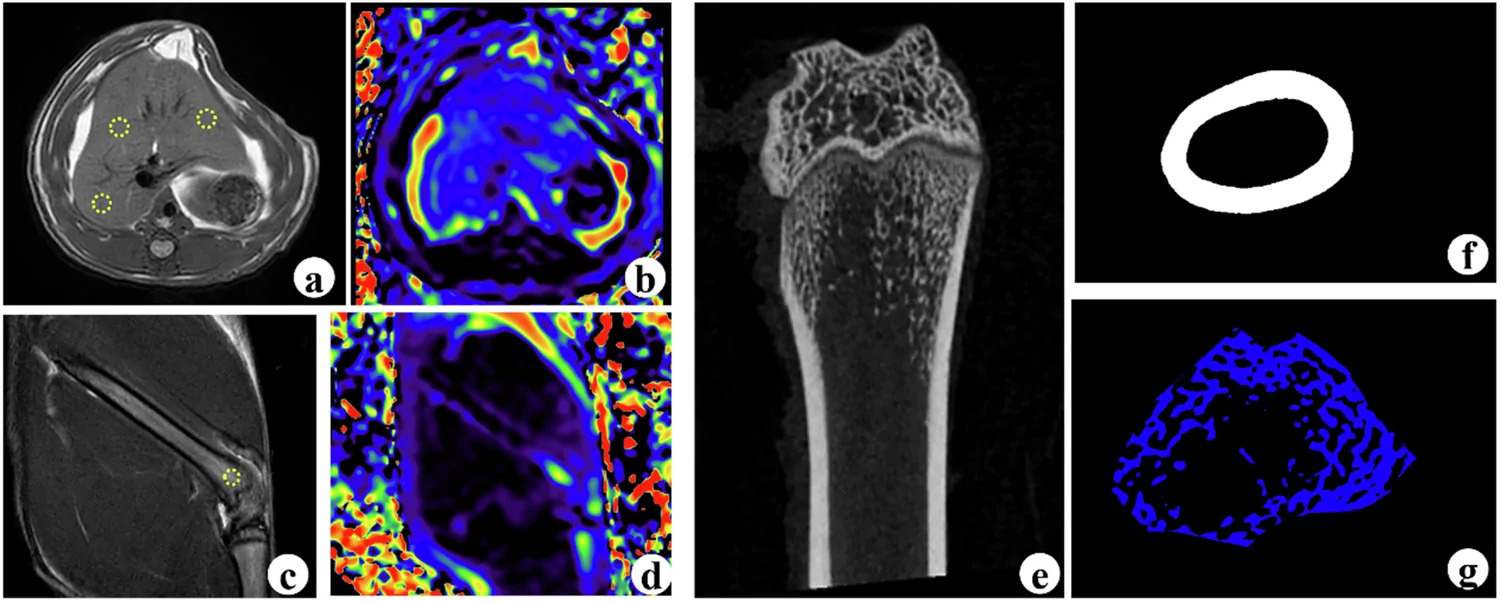
Magnetic resonance imaging of the liver and right femur, and micro-CT images of the femur. **a** T2WI of the liver; **b** PDFF image of the liver; **c** T2WI of the right femur; **d** PDFF image of the right femur. **e**–**g** Micro-CT images of the femur. Micro-CT, Micro-computed tomography; PDFF, Proton density fat fraction; T2DM, Type 2 diabetes mellitus; T2WI, T2-weighted images

For liver imaging, axial images were acquired using a 6-echo sequence with the following parameters: repetition time/echo time = 17.0/1.31 ms; flip angle = 3°; slice thickness = 1.5 mm; acquisition matrix = 48 × 47; number of slices = 10; interslice gap = 0 mm; field of view = 70 × 70 ×  15 mm^3^; sensitivity encoding acceleration factor = 2; and scan time = 14 s. Three circular ROIs were placed on the liver T2-weighted images (Fig. 2a), excluding vessels, bile ducts, and the liver margin. To ensure anatomical consistency across multiparametric measurements, the same ROIs were duplicated onto the co-registered proton density fat fraction (PDFF, Fig. 2b) generated from the mDIXON Quant imaging. Mean PDFF (%) values from the three ROIs were calculated for subsequent analysis.

For femoral imaging, sagittal images were acquired using a 6-echo sequence with the following parameters: repetition time/echo time = 7.2/1.32 ms; flip angle = 3°; slice thickness = 2 mm; acquisition matrix = 48 × 47; number of slices = 5; interslice gap = 0 mm; field of view =  70 × 70 × 10 mm^3^; sensitivity encoding acceleration factor = 2; and scan time = 14 s. Two circular ROIs were placed on the femoral T2-weighted images (Fig. 2c), excluding cortical bone. These ROIs were duplicated onto the corresponding PDFF (Fig. 2d) to maintain anatomical consistency. Mean PDFF (%) values from the two ROIs were calculated for subsequent analysis.

### Lipid metabolism and biochemical assessments

After decapitation, the thoracic cavity was rapidly opened to expose the heart, and blood was collected by ventricular puncture. The blood samples were placed in anticoagulant tubes, gently mixed, and centrifuged at 8,000 rpm for 15 min at 4 °C (Sigma 3-18KHS centrifuge, Sigma Laborzentrifugen GmbH, Osterode am Harz, Germany). The supernatant serum was collected and aliquoted into 1.5-mL centrifuge tubes.

Lipid metabolism and biochemical parameters included total cholesterol, triglycerides, high-density lipoprotein cholesterol, low-density lipoprotein cholesterol, alanine aminotransferase (ALT), aspartate aminotransferase (AST), serum calcium, serum magnesium and serum phosphorus. These parameters were measured using an automatic biochemical analyzer. Instrument operation strictly followed the manufacturer’s calibration and quality control procedures to ensure the accuracy and reliability of the experimental data.

### Micro-computed tomography (micro-CT) protocol and image analysis

All right femur specimens were scanned using a SkyScan 1176 micro-CT system (Bruker, Kontich, Belgium). The scanning parameters were as follows: voxel size = 16.62 μm, x-ray voltage = 65 kV, and current = 385 μA.

Image reconstruction and analysis were performed using NRecon 1.7 and CTAn 1.17 software, and three-dimensional reconstructions were generated using CTvox software. Trabecular bone microarchitecture was analyzed in the distal femur using 100 consecutive slices (1.66 mm) starting proximally from the distal femoral growth plate. The cortical bone structure was assessed at the femoral midshaft using 100 consecutive slices (0.83 mm) located 650 slices (10.69 mm) above the distal growth plate (Fig. 2e–g). Quantitative parameters analyzed using the Custom Analysis program (CTAn; SkyScan) included BMD, bone volume fraction (BV/TV), trabecular thickness, trabecular number, trabecular separation, structure model index, cortical thickness, total cortical bone area, and cortical porosity (Ct.Po).

### Biomechanical analysis

The right femur specimens were thawed at room temperature for approximately 30 min prior to testing. Residual soft tissues, muscles, and ligaments surrounding each sample were carefully removed using surgical forceps and scissors. Biomechanical measurements were performed at the mid-diaphysis of the right femur. A three-point bending test was conducted using a BOSE 3,200 biomechanical testing system (Bose Corporation, Eden Prairie, USA). Each bone was positioned horizontally on two supporting points with a span distance of 10 mm, with the proximal end placed beneath the greater trochanter and the distal end resting on the support. A vertical load was applied at the midshaft of the femur at a constant displacement rate of 0.05 mm/s until complete fracture occurred. The force-displacement curve was recorded in real-time during the test. The following biomechanical parameters were calculated from the force-displacement curve: ultimate load, elastic stiffness and total work to failure.

### Masson’s staining and analysis

Femurs were fixed in paraformaldehyde, decalcified with ethylenediaminetetraacetic acid, and embedded in paraffin. Serial 4-μm sections were prepared. After dewaxing and antigen retrieval, the sections were blocked with 5% bovine serum albumin, incubated with a collagen type I primary antibody, and then with a horseradish peroxidase-labeled secondary antibody. Color development was performed using 3,3′-diaminobenzidine, followed by hematoxylin counterstaining. Images were captured using an optical microscope and imported into ImageJ software for background correction, color deconvolution, and threshold segmentation. The area occupied by bone collagen fibers and their corresponding percentage were calculated from the Masson-stained tissue sections.

### Enzyme-linked immunosorbent assay (ELISA) and real-time quantitative polymerase chain reaction (PCR) for bone metabolism markers and AGEs

Shred the rats’ tibia and fibula, and the content of procollagen I N-terminal propeptide (PINP), carboxy-terminal peptide (CTX), and AGEs were measured using the ELISA method as the following procedures (ELISA kits all obtained from Zhuocai Biotechnology Co., Ltd, Shanghai, China): wash the tissue with pre-chilled phosphate-buffered saline (0.01 M, pH 7.4) to remove residual blood; after weighing, homogenize the tissue; combine the homogenized tissue with an appropriate volume of phosphate-buffered saline; add protease inhibitor to phosphate-buffered saline; transfer to a glass homogenizer and homogenize thoroughly on ice; centrifuge the homogenate at 5,000 × *g* for 5–10 min, then collect the supernatant for analysis. The target antibody is immobilized on a solid-phase carrier in a 96-well microplate. Standard samples or test specimens are added to the wells, where the target antibody binds to the carrier. Horseradish peroxidase-labeled antibodies are then added. Unbound antibodies are washed off, followed by thorough rinsing and the addition of TMB substrate to develop color. TMB is catalyzed by peroxidase to form a blue color, which then turns yellow under acidic conditions. The color intensity is positively correlated with the target concentration in the sample. Absorbance (optical density value) is measured at 450 nm using a microplate reader to determine the sample concentration.

The contents of osteopontin (OPN) and osteocalcin (OCN) relative messenger ribonucleic acid (mRNA) expression were measured using the real-time quantitative PCR method as follows. Shred the rats’ tibia and fibula, extracted total RNA, quantified and identified RNA, followed by RNA reverse transcription. Finally, mRNA was quantified using real-time quantitative PCR as follows.

### Statistical analysis

Continuous variables were assessed for normality using the Shapiro–Wilk test. Normally distributed data were presented as means ± standard deviations and were compared using independent-samples *t*-tests. Non-normally distributed or ordinal data were presented as medians with interquartile ranges and compared using the Wilcoxon rank-sum test.

Spearman correlation analysis was used to evaluate pairwise associations among hepatic PDFF, femoral PDFF, and the area and organization of femoral collagen fibers.

Statistical analyses were performed using SPSS (version 26.0; IBM) and GraphPad Prism (GraphPad Software, LLC, version 8.4.2). All tests were two-sided, and *p* < 0.05 was considered statistically significant.

## Results

### General characteristics of T2DM rats at 2 weeks of modeling

**Fig. 3:**
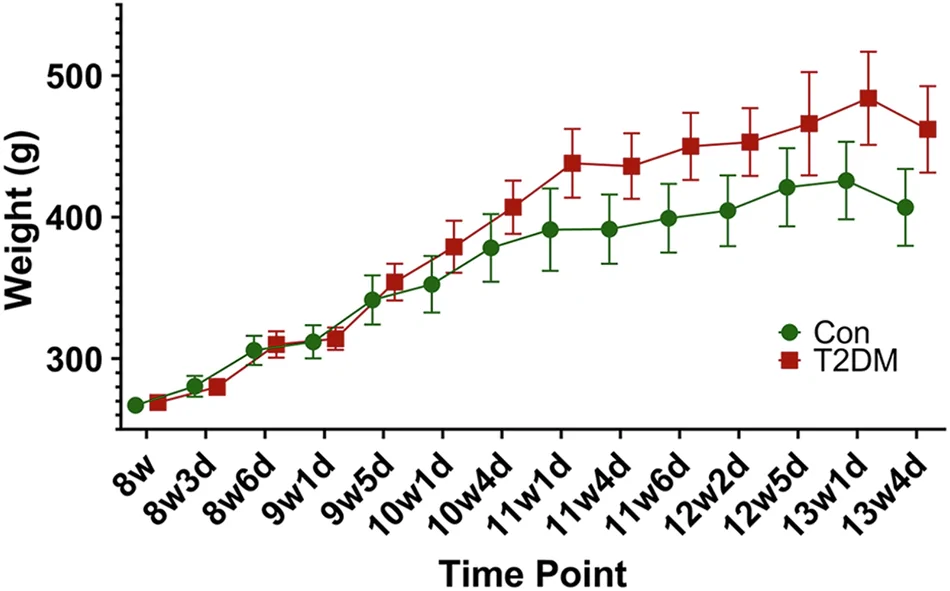
Line chart exhibiting changes in body weight of Sprague-Dawley rats in the control and T2DM groups over time. T2DM, Type 2 diabetes mellitus

The body weight of SD rats in the T2DM group was higher than that in the control group starting at 10 weeks and 1 day of age (Fig. 3). Serum total cholesterol, ALT, and ALT/AST levels were increased in the T2DM group compared with the control group (Table 1).

**Table 1:** Differences in the general characteristics of SD rats in the control group and the T2DM group

Characteristic	Control group (*n* = 6)	Diabetes group (*n* = 6)	*p-*value
**TC (mmol/L)**	**1.31** ± **0.36**	**2.34** ± **0.97**	**0.048**
TG (mmol/L)	0.67 ± 0.31	0.70 ± 0.15	0.861
HDL-C (mmol/L)	0.37 ± 0.17	0.41 ± 0.28	0.759
LDL-C (mmol/L)	0.38 ± 0.09	0.66 ± 0.39	0.142
**ALT (U/L)**	**36.50 (31.25, 40.50)**	**137.50 (83.00, 477.75)**	**0.002**
AST (U/L)	119.50 (99.25, 237.00)	241.00 (169.50, 823.25)	0.065
**ALT/AST**	**0.27** ± **0.11**	**0.54** ± **0.16**	**0.006**
Calcium (mmol/L)	2.83 ± 0.14	2.72 ± 0.14	0.216
Magnesium (mmol/L)	1.60 ± 0.09	1.44 ± 0.17	0.069
Phosphorus (mmol/L)	2.62 ± 0.66	3.00 ± 0.36	0.245
DXA parameters			
**Fat mass (g)**	**72.20** ± **13.28**	**105.33** ± **19.21**	**0.006**
Muscle mass (g)	338.28 ± 17.42	324.72 ± 14.45	0.173
**Fat in tissue percentage (%)**	**17.47** ± **2.16**	**24.35** ± **2.87**	**0.001**
MRI parameters			
**Hepatic PDFF value**	**2.05 (1.23, 2.51)**	**14.62 (3.13, 29.65)**	**0.002**
Femoral PDFF value	7.99 (2.20, 16.10)	24.19 (9.61, 45.02)	0.093
micro-CT parameters			
Cancellous			
BMD (g/cm^3^)	0.62 ± 0.01	0.60 ± 0.03	0.185
BV/TV (%)	43.96 (26.78, 46.32)	25.42 (13.94, 36.27)	0.200
Tb.Th (mm)	0.17 ± 0.02	0.16 ± 0.04	0.406
Tb.N (1/mm)	2.16 ± 0.58	1.62 ± 0.47	0.108
Tb.Sp (mm)	0.42 (0.34, 0.64)	0.61 (0.51, 0.93)	0.150
SMI	0.38 (0.28, 1.50)	1.46 (0.76, 1.92)	0.262
Cortex			
BMD (g/cm^3^)	1.31 ± 0.03	1.31 ± 0.02	0.754
BV/TV (%)	94.02 ± 4.03	93.56 ± 3.42	0.836
Ct.Th (mm)	0.60 ± 0.02	0.59 ± 0.05	0.726
Ct.Ar (mm2)	50.43 ± 2.08	52.67 ± 4.62	0.306
Ct.Po (%)	5.98 ± 4.03	6.44 ± 3.42	0.836
Biomechanical analysis			
Ultimate load (N)	117.31 ± 6.50	122.65 ± 16.29	0.482
Elastic stiffness (N/mm)	74.12 ± 12.84	69.71 ± 14.19	0.585
TWTF (mJ)	73.35 (64.22, 101.40)	68.59 (55.50, 79.25)	0.310
Femoral collagen fibers			
Area (%)	33.00 ± 5.62	28.21 ± 11.05	0.367
Dispersion (°)	24.66 ± 6.04	25.79 ± 7.49	0.780
**Goodness**	**0.91** ± **0.05**	**0.80** ± **0.08**	**0.022**

*ALT* Alanine aminotransferase, *AST* Aspartate aminotransferase, *BMD* Bone mineral density, *BV/TV* Bone volume fraction, *Ct.Ar* Total cortical bone area, *Ct.Po* Cortical porosity, *Ct.Th* Cortical thickness, *HDL-C* High-density lipoprotein cholesterol, *LDL-C* Low-density lipoprotein cholesterol, *PDFF* Proton density fat fraction, *SMI* Structure model index, *SD* Sprague-Dawley, *T2DM* Type 2 diabetes mellitus, *TC* Total cholesterol, *TG* Triglycerides, *Tb.N* Trabecular number, *Tb.Sp* Trabecular separation, *Tb.Th* Trabecular thickness, *TWTF* Total work to failure. Bold rows/values indicated statistically significant differences between the control group and the Diabetes group (*p* < 0.05)

### Adipose tissue distribution

Whole-body composition analysis revealed that fat mass and body fat percentage derived from DXA were higher in the T2DM group (Table 1). The hepatic PDFF values were higher in the T2DM group than in the control group (Table 1). No significant differences were observed in femoral PDFF between groups (Table 1). Within 2 weeks of T2DM onset, SD rats in the T2DM group demonstrated increased femoral PDFF, although the difference did not reach statistical significance.

### Bone microstructure based on micro-CT and biomechanical analysis

Cancellous BV/TV and trabecular number in T2DM were lower than the control group, trabecular separation and structure model index in T2DM were higher than the control group, and total cortical bone area and Ct.Po of in T2DM were higher than the control group, while the differences were not statistically significant (Table 1). Ultimate load in T2DM was higher than the control group, elastic stiffness and total work to failure were lower than the control group, while the differences were not statistically significant (Table 1).

### Bone metabolism markers and AGEs

**Fig. 4:**
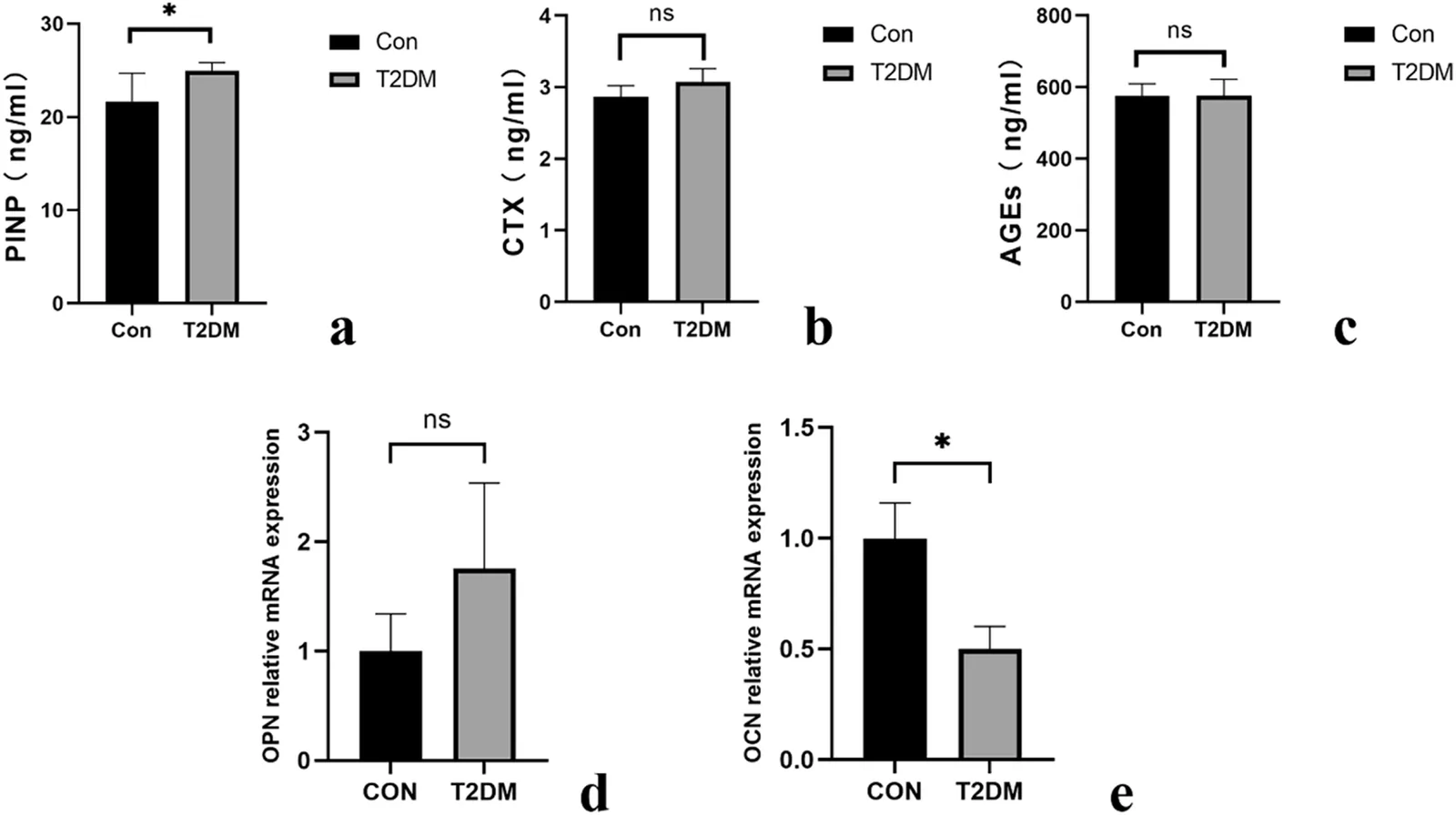
Comparison of bone metabolism markers and AGEs levels between control and T2DM groups. **a** PINP between control and T2DM groups; **b** CTX between control and T2DM groups; **c** AGEs between control and T2DM groups; **d** OPN relative mRNA expression between control and T2DM groups; **e** OCN relative mRNA expression between control and T2DM groups. AGEs, Advanced glycation end products; T2DM, Type 2 diabetes mellitus; PINP, Procollagen I N-terminal propeptide; CTX, Carboxy-terminal peptide; OPN, Osteopontin; OCN, Osteocalcin; mRNA, messenger ribonucleic acid

There was no difference in calcium, magnesium and phosphorus between the control and T2DM group (*p* = 0.216, *p* = 0.069, and *p* = 0.245, Table 1). PINP of the T2DM group was higher than the control group (*p* = 0.043, Fig. 4a), and there was no difference of CTX between the control and T2DM group (*p* = 0.071, Fig. 4b). There was no difference of AGEs between the control and T2DM group (*p* = 0.963, Fig. 4c). There was no difference of OPN relative mRNA expression between the control and T2DM group (*p* = 0.413, Fig. 4d), and OCN relative mRNA expression of the T2DM group was lower than the control group (*p* = 0.046, Fig. 4e).

### Osteo-collagenous fibers analysis

**Fig. 5:**
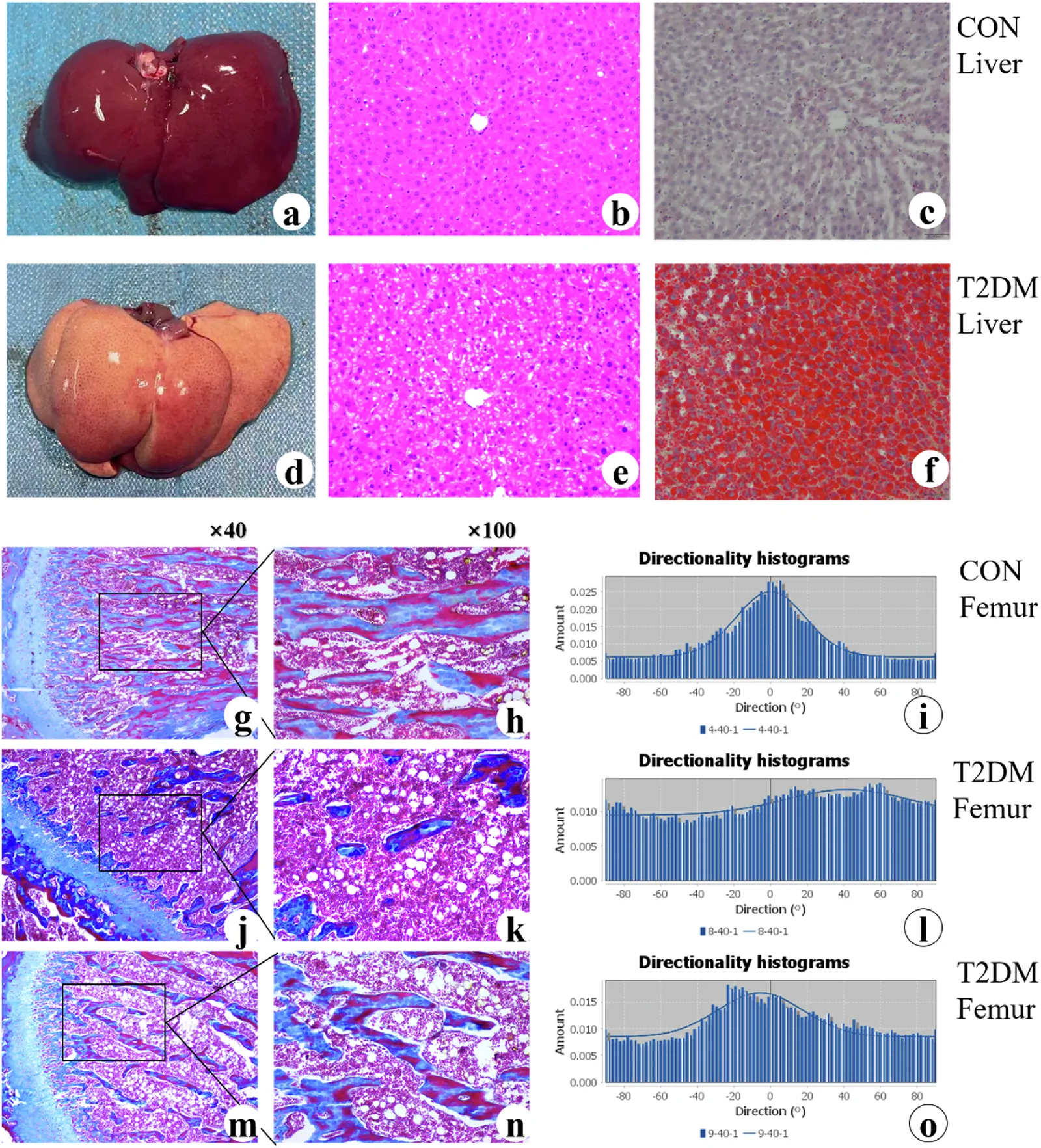
Gross specimens, hematoxylin and eosin staining, and oil red staining of the liver and Masson’s staining of the distal femur in the control group and the T2DM group. **a** Gross liver specimen of the control group; **b** hematoxylin and esosin staining of the liver in the control group; **c** oil red staining of the liver in the control group; **d** gross liver specimen of the T2DM group; **e** hematoxylin and esosin staining of the liver in the T2DM group; **f** oil red staining of the liver in the T2DM group; **g** Masson’s staining of the femur in the control group (×40); **h** Masson’s staining of the femur in the control group (×100); **i** histogram showing the distribution of collagen fiber orientation in the femur of the control group; **j** Masson’s staining of the femur in the T2DM group (×40); **k** Masson’s staining of the femur in the T2DM group (×100); **l** histogram showing the distribution of collagen fiber orientation in the femur of the T2DM group; **m** Masson’s staining of the femur in the T2DM group (×40); **n** Masson’s staining of the femur in the T2DM group (×100); **o** histogram showing the distribution of collagen fiber orientation in the femur of the T2DM group. T2DM, Type 2 diabetes mellitus

The fat deposition of liver and right femur in T2DM was increased compared to the control group (Fig. 5). Area of right femur collagenous fibers in T2DM was lower than the control group, while the difference was not statistically significant (Table 1 and Fig. 5). Femoral collagen fiber goodness in T2DM was lower than the control group (*p* = 0.022, Table 1 and Fig. 5).

**Fig. 6:**
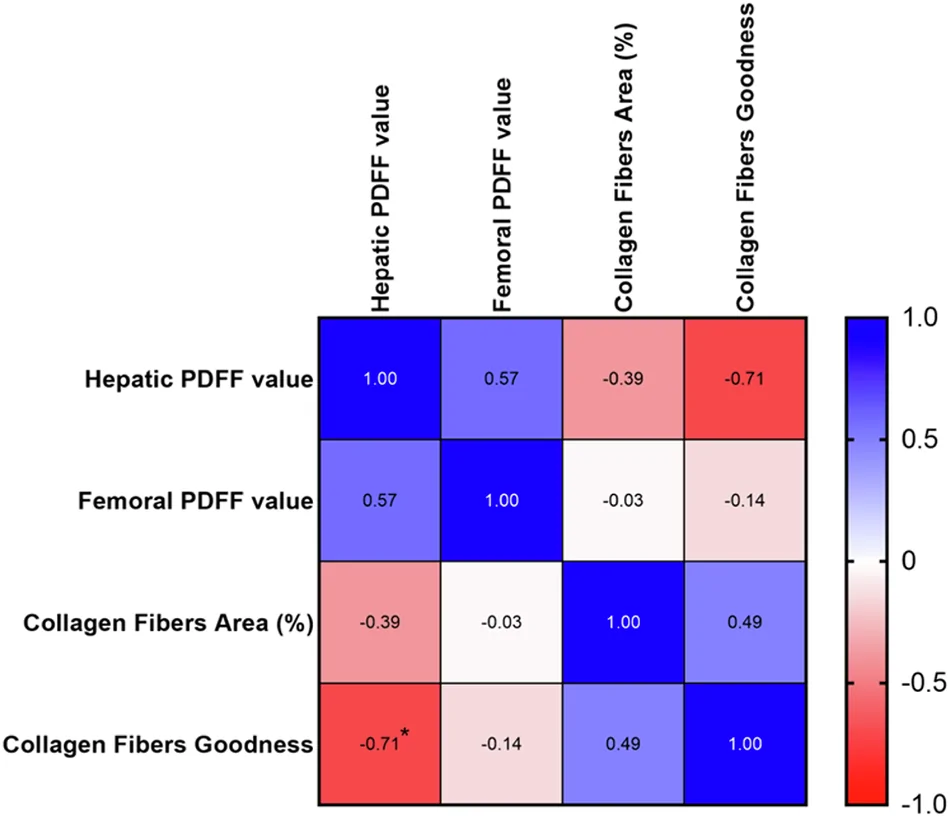
Correlations among hepatic PDFF, femoral PDFF, and the area and goodness of femoral collagen fibers. PDFF, Proton density fat fraction

Femoral collagen fiber goodness was negatively correlated with hepatic PDFF values (*r* = -0.709, *p* = 0.012, Fig. 6).

## Discussion

Following an adaptation period and 1 week of high-fat diet (45% fat) feeding, SD rats assigned to diabetes induction exhibited a progressive increase in body weight compared with the control group. After successful establishment of the T2DM model, comparative analyses performed within 2 weeks (DXA and serum biochemistry) demonstrated significantly elevated total cholesterol level, fat mass, and body fat percentage in the T2DM group. Diabetes is associated with numerous complications, including obesity and hypercholesterolemia [18]. A 2024 *Lancet Public Health* study reported that overweight and obesity are crucial determinants strengthening the association between insulin resistance and diabetes [19]. Consistent with these findings, the body weight of rats in the T2DM group was significantly higher than that in the control group before successful modeling.

Within 2 weeks of T2DM onset, SD rats in the T2DM group exhibited elevated ALT, AST, ALT/AST ratio, and hepatic PDFF value compared with the control group. Among these parameters, ALT, the ALT/AST ratio, and hepatic PDFF showed statistically significant differences. Previous studies have demonstrated that hepatic PDFF is an independent risk factor for insulin resistance [20]. A study published in *Radiology* in 2025 reported higher ALT and AST levels in T2DM than in controls, and that severe hepatic steatosis (PDFF ≥ 25%) was associated with increased odds of prediabetes (odds ratio, 3.49; 95% confidence interval (CI): 1.35, 9.02; *p* = 0.010) and T2DM (odds ratio, 3.45; 95% CI: 1.30, 9.13; *p* = 0.010) [4]. Prior studies have demonstrated that hepatic steatosis is associated with a higher risk of glucose metabolism disorders, which aligns with our findings.

Micro-CT analysis revealed no significant differences in femoral cortical bone parameters, including BMD, BV/TV, cortical thickness, total cortical bone area and Ct.Po, while the Ct.Po slightly increased in T2DM. For trabecular bone, BV/TV, trabecular thickness, and trabecular number were reduced, whereas trabecular separation and structure model index were increased. However, these changes were not statistically significant either. Although the area occupied by femoral collagen fibers was reduced in the T2DM group, this difference was not statistically significant. In contrast, the organization of femoral collagen fibers was significantly poorer in the T2DM group than in the control group. Given that the only significant bone-related change in early T2DM was poorer collagen fiber organization (a matrix-level property rather than a microstructural parameter), our findings suggest that deterioration of bone quality at the matrix/material level may occur before overt trabecular microstructural alterations. And, although the increase in Ct.Po in T2DM rats did not reach statistical significance, the observed mild elevation is consistent with the possibility that Ct.Po begins to increase during the early stage of T2DM, potentially serving as an early manifestation of bone microstructural deterioration, which needs to be confirmed. This may be related to the higher metabolic activity of trabecular bone and the association between increased cortical porosity and fracture risk in T2DM [21]. Biomechanical analysis revealed a slight (non-significant) increase in ultimate load and decrease in total work to failure in T2DM rats, indicating that bone biomechanical properties—particularly torsional toughness—are already impaired in the early stages of T2DM. This finding is compatible with the observed disorganization of collagen fibers, as collagen integrity is essential for bone resistance to torsion and energy absorption.

Previous studies have shown that serum calcium levels in patients with T2DM are typically at the lower limit of the normal range or slightly decreased [22], with decreased serum magnesium levels occurring in 10%–30% of cases, and serum phosphorus levels generally remain within the normal range or exhibit a mild elevation [23]. In the present study, the levels of serum calcium and magnesium were slightly decreased, while the level of serum phosphorus was slightly increased; however, no significant statistical differences were observed, which is consistent with previous research findings. PINP of the T2DM group was higher than that of the control group, and no difference in CTX between the control and T2DM groups. PINP and CTX, as classical bone turnover markers, exhibit a characteristic dissociation pattern early in the course of T2DM. Previous studies have shown that compared to healthy controls, serum PINP levels in newly diagnosed or T2DM patients with a duration of less than 5 years often show no significant increase or even a slight decrease, while CTX demonstrates a more pronounced declining trend, resulting in an elevated PINP/CTX ratio [24]. In the present study, PINP was elevated while CTX showed no significant difference, which diverges from prior research findings. In the early stage of T2DM, osteoblastic activity may increase in a compensatory response, leading to elevated PINP levels. OCN relative mRNA expression of the T2DM group was lower, and there was no difference in OPN relative mRNA expression between the control and T2DM group. Changes in the expression of OPN and OCN reflect a deep-seated dysregulation of the bone-energy metabolism axis. In patients with T2DM, OCN levels are often significantly reduced, indicating suppressed bone formation [25]. In contrast, OPN is persistently upregulated from the early stages of T2DM. It serves not only as a regulator of bone mineralization but also as a critical link between metabolism and inflammation. In tissues such as adipose tissue, liver, and blood vessels, the expression of OPN is regulated by inflammatory factors (*e.g*., tumor necrosis factor-α, Interleukin-6) and oxidative stress induced by hyperglycemia [26]. The upregulation of OPN is not merely a response of bone metabolism but also a manifestation of systemic metabolic inflammation within the skeletal system, marking a critical transition from “bone metabolic disorder” to “bone-vascular comorbidity”.

Although no statistically significant difference in the area occupied by femoral collagen fibers was observed between the T2DM and control groups, it was slightly reduced in the T2DM group compared with the control group. Within 2 weeks of T2DM onset, the femoral collagen fiber organization showed strong negative correlations with the hepatic PDFF value. The femoral collagen fiber organization reflects collagen fiber orientation, with values closer to 1 indicating a more ordered alignment and lower values indicating greater disorganization. Accumulation of AGEs has been identified as a major contributor to collagen fiber disorganization in diabetic bone [7, 27]. In the present study, however, AGE levels in the tibia and fibula did not significantly differ between groups, suggesting that early-stage T2DM may not yet induce measurable AGE accumulation in bone. We speculated that hepatic fat accumulation may disrupt bone collagen fiber organization through AGE-independent molecular mechanisms, which warrant further investigation.

This study had some limitations. First, the sample size was relatively small, with only 6 SD rats per group, which may limit statistical power and generalizability. Second, this study did not obtain DXA-derived indices, such as trabecular bone score, bone strain index, and hip structural analysis, which could provide complementary radiological information on bone quantity and quality *in vivo*. Future studies using these non-invasive assessment tools would help bridge the gap between preclinical observations and clinical evaluation of bone quality in patients with T2DM. Third, an MRI was performed using a single 3.0-T MRI system (with the mDIXON Quant). The use of higher-field MRI systems (*e.g*., 7- or 9.4-T magnets), which could provide enhanced signal-to-noise ratio and spectral resolution, was not explored. Fourth, we only measured serum calcium, magnesium and phosphorus, without detecting vitamin D levels. Vitamin D deficiency is highly prevalent among patients with T2DM, and mineral and vitamin D metabolism are interconnected, collectively forming a complex metabolic network. In the present study, there were no significant differences in serum calcium, magnesium and phosphorus, suggesting that vitamin D levels remain unchanged in early-stage T2DM. In the future, we will incorporate serum vitamin D levels for further research. Finally, the study focused on early-stage changes within 2 weeks of T2DM induction, and longer-term progression remains to be investigated.

In early-stage T2DM, bone damage exhibits a temporal sequence: organic matrix impairment (reduced and disorganized collagen fibers) precedes inorganic matrix damage. Microstructural deterioration is more advanced in cancellous bone compared to cortical bone. These bone alterations coincide with systemic metabolic disturbances, including adipose tissue redistribution and hepatic steatosis, which correlate with both liver dysfunction and the degree of bone collagen disorganization. During the diagnostic workup of T2DM, an elevated liver MRI-derived PDFF may indicate compromised bone quality, warranting bone quality screening with DXA-derived parameters such as trabecular bone score, hip structural analysis, and bone strain index. Furthermore, therapeutic interventions aimed at ameliorating adipose and hepatic dysfunction in T2DM may help restore bone collagen matrix homeostasis.

## Data Availability

The datasets used and/or analyzed during the current study are available from the corresponding author on reasonable request.

## Abbreviations

AGEs: Advanced glycation end products
ALT: Alanine aminotransferase
AST: Aspartate aminotransferase
BMD: Bone mineral density
BV/TV: Bone volume fraction
Ct.Po: Cortical porosity
CTX: Carboxy-terminal peptide
DXA: Dual-energy x-ray absorptiometry
ELISA: Enzyme-linked immunosorbent assay
Micro-CT: Micro-computed tomography
mRNA: Messenger ribonucleic acid
OCN: Osteocalcin
OPN: Osteopontin
PCR: Polymerase chain reaction
PDFF: Proton density fat fraction
PINP: Procollagen I N-terminal propeptide
ROI: Region of interest
SD: Sprague-Dawley
T2DM: Type 2 diabetes mellitus
